# Three-Year Trend in *Escherichia coli* Antimicrobial Resistance among Children’s Urine Cultures in an Italian Metropolitan Area

**DOI:** 10.3390/children8070597

**Published:** 2021-07-14

**Authors:** Luca Pierantoni, Laura Andreozzi, Simone Ambretti, Arianna Dondi, Carlotta Biagi, Francesco Baccelli, Marcello Lanari

**Affiliations:** 1 Division of Pediatric Emergency, IRCCS Azienda Ospedaliero-Universitaria di Bologna, 40138 Bologna, Italy; luca.pierantoni@aosp.bo.it (L.P.); arianna.dondi@gmail.com (A.D.); carlotta.biagi@aosp.bo.it (C.B.); marcello.lanari@unibo.it (M.L.); 2 Specialty School of Paediatrics—Alma Mater Studiorum, Università di Bologna, 40138 Bologna, Italy; f.baccelli92@gmail.com; 3 Unit of Clinical Microbiology, IRCCS Azienda Ospedaliero-Universitaria di Bologna, 40138 Bologna, Italy; simone.ambretti@aosp.bo.it

**Keywords:** bacterial infections, urinary tract infections, *Escherichia coli*, antimicrobial resistance, children, amoxicillin clavulanate, multidrug resistance, extensively drug resistant, ESBL

## Abstract

Urinary tract infections (UTIs) are among the most common bacterial infections in children, and *Escherichia coli* is the main pathogen responsible. Several guidelines, including the recently updated Italian guidelines, recommend amoxicillin-clavulanic acid (AMC) as a first-line antibiotic therapy in children with febrile UTIs. Given the current increasing rates of antibiotic resistance worldwide, this study aimed to investigate the three-year trend in the resistance rate of *E. coli* isolated from pediatric urine cultures (UCs) in a metropolitan area of northern Italy. We conducted a retrospective review of *E. coli*-positive, non-repetitive UCs collected in children aged from 1 month to 14 years, regardless of a diagnosis of UTI, catheter colonization, urine contamination, or asymptomatic bacteriuria. During the study period, the rate of resistance to AMC significantly increased from 17.6% to 40.2% (*p* < 0.001). Ciprofloxacin doubled its resistance rate from 9.1% to 16.3% (*p* = 0.007). The prevalence of multidrug-resistant *E. coli* rose from 3.9% to 9.2% (*p* = 0.015). The rate of resistance to other considered antibiotics remained stable, as did the prevalence of extended spectrum beta-lactamases and extensively resistant *E. coli* among isolates. These findings call into question the use of AMC as a first-line therapy for pediatric UTIs in our population, despite the indications of recent Italian guidelines.

## 1. Introduction

Urinary tract infections (UTIs) are one of the most common types of bacterial infections in the pediatric population [1]. In 2011, UTIs accounted for 844 per 100,000 visits in pediatric emergency departments (PEDs) in the USA according to the Nationwide Emergency Department Sample [2]. Within the sixth year of life, 3–7% of girls and 1–2% of boys will be diagnosed with a UTI and about 12–30% of these children will develop recurrent UTIs [1].

*Escherichia coli* is the most frequent pathogen responsible, causing approximately 80–90% of UTIs in children [1]. In febrile UTIs, empiric treatment should be started while waiting for antibiotic susceptibility results. A rational antimicrobial approach to UTIs should consider the predominant uropathogens isolated in acute community-acquired infections and local sensitivity patterns [3,4]. The importance of the first-line therapy is reflected by the risk of sepsis and renal scarring or abscesses, which may complicate a UTI.

Several guidelines, including the recently updated Italian guidelines, recommend amoxicillin-clavulanic acid (AMC) as a first-line antibiotic therapy in children with febrile UTIs [4,5]. In Italy, the choice of AMC as a first-line therapy for many UTIs is based on several studies conducted over the last 10 years on cohorts of Italian children. These studies recorded a rising concern about the increasing incidence of *E. coli* extended spectrum beta-lactamases (ESBL)-producing strains [6,7] that may remain susceptible to clavulanic acid combinations. In these studies, the rate of *E. coli* resistance to AMC appeared to be limited (9% to 15%) [8,9].

Recently, an increasing resistance rate of *E. coli* strains to several antibiotics, including AMC, was reported by authors from several countries, resulting in growing concerns among clinicians [1].

The aim of our study was to investigate the trend of the in vitro resistance rate of *E. coli*, isolated from pediatric urine cultures (UCs), to antibiotics over a period of three years in a metropolitan area of northern Italy.

## 2. Materials and Methods

We conducted a retrospective review of UCs collected between January 2017 and December 2019 from a pediatric population in the largest metropolitan area in the Emilia-Romagna region, located in northern Italy, covering about 1 million people and including PEDs and pediatric wards in three different hospitals: S. Orsola University Hospital and Maggiore Hospital, both located in Bologna, and S. Maria della Scaletta Hospital in Imola.

We obtained the *E. coli*-positive UCs of patients aged from 1 month to 14 years from the database of the laboratory of Clinical Microbiology of S. Orsola University Hospital. This laboratory analyzes all UCs collected in the metropolitan area from the PEDs, the pediatric wards of the three hospitals, and the community health centers within the Italian healthcare system.

We included non-repetitive UCs with at least 10,000 colony-forming units per milliliter, regardless of the collection method used (clean catch method, urine bag collection, or bladder catheterization). All positive urinary cultures were evaluated, regardless of whether they had a diagnosis of upper or lower UTI, catheter colonization, urine contamination, or asymptomatic bacteriuria.

The study was conducted in accordance with the Declaration of Helsinki, and the protocol was approved by the local Ethics Committee (Comitato Etico Area Vasta Emilia Centro, AVEC; project identification code: 882/2020/Oss/AOUBo).

### 2.1. Laboratory Methods

All urine samples were processed by the WASPlab bacteriology platform (Copan, Brescia, Italy). Quantitative urine cultures were performed with standard techniques using CHROMagar Orientation media (KIMA Meus, Padova, Italy). Plates were streaked (1 microliter loop) and incubated at 35–37 °C for 16 h. All isolates were identified using chromogenic media or MALDI-TOF mass spectrometry (Vitek-MS, Biomerieux, Marcy L’Etoile, France), as recommended by the manufacturers.

Antimicrobial susceptibility testing was performed using the VITEK2 semiautomated system (Biomerieux, Marcy L’Etoile, France). The following antimicrobial drugs were tested against Gram-negative strains: ampicillin, AMC, piperacillin/tazobactam, cefotaxime, ceftazidime, ertapenem, meropenem, amikacin, gentamicin, cotrimoxazole, nitrofurantoin, fosfomycin, and ciprofloxacin. Susceptibility results for all the tested antimicrobials were interpreted following the EUCAST (European Committee on Antimicrobial Susceptibility Testing) clinical breakpoints.

*E. coli* which hydrolyzes penicillin, and first- to third-generation cephalosporins and aztreonam were defined phenotypically as ESBL.

Criteria for the definitions of multidrug-resistant (MDR) and extensively resistant (XDR) *E. coli* were identified according to the combined guidelines of the European Centre for Disease Prevention and Control (ECDC) and the Centers for Disease Control and Prevention (CDC). MDR *E. coli* were defined as non-susceptible to one agent in three antimicrobial categories. *E. coli* were considered XDR if non-susceptible to one agent in all but two categories [10].

### 2.2. Statistical Analysis

Descriptive analyses were reported as number and relative percentages if categorical, while quantitative variables were presented as means and standard deviations (SDs) or medians and interquartile ranges (IQRs) as appropriate. Annual resistance rates were compared with the Pearson Chi-square test. The level of statistical significance was set at *p* < 0.05. The analysis was performed with SPSS Statistics V25 for Windows.

## 3. Results

**Figure 1:**
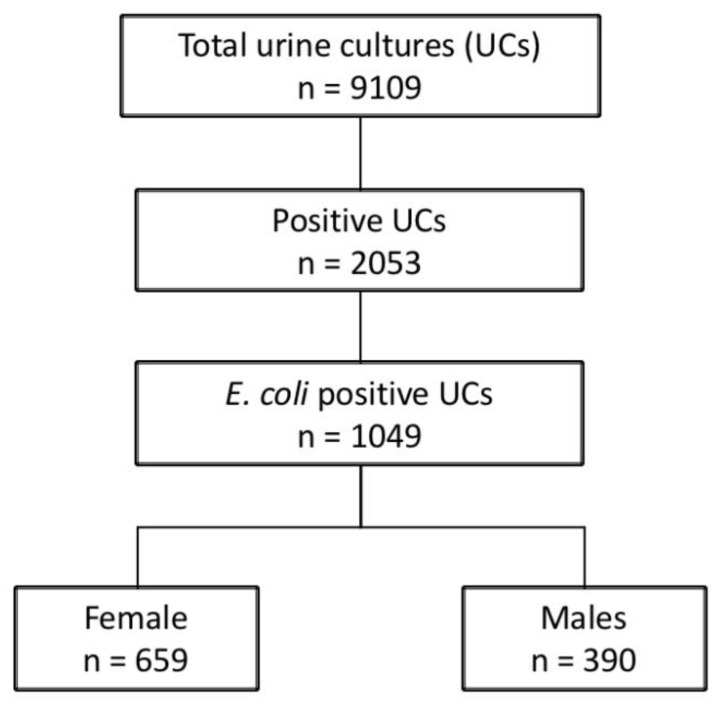
Patient flow chart.

As reported in Figure 1, during the study period 1049 *E. coli* positive UCs were collected (363, 360, and 326 over the calendar years 2017, 2018, and 2019, respectively). UCs were obtained from female children in 659 (62.8%) cases.

Before the age of three, the male/female ratio was slightly below one (M/F = 0.8). Over the age of three years, female children accounted for 77.6% of the total patients, with a male/female ratio of 0.3. The median (interquartile range, IQR) age at UC collection was 1.22 (0.49–4.56) years. The UCs were collected in children <3, 3–5, 6–10, >10 years in 687 (65.5%), 162 (15.4%), 148 (14.1%), and 52 (5%) cases, respectively. The samples were collected in community health centers in 611 (58.2%) cases, in PEDs in 185 (17.6%) cases, and in pediatric hospital wards in 253 (24.1%) cases.

Over the entire study period, 296 (28.2%) *E. coli* isolates were found to be resistant to AMC, while 238 (22.7%) cases were found to be resistant to cotrimoxazole. Resistance to ciprofloxacin, piperacillin/tazobactam, cefotaxime, ceftazidime, and gentamicin was reported in 122 (11.6%), 62 (5.9%), 61 (5.8%), 60 (5.7%), and 48 (4.6%) isolates, respectively. The rates of resistance to amikacin, fosfomycin, and nitrofurantoin were found to be almost zero over the entire study period (0.6%, 0.6%, and 0.3%, respectively). The resistance rates among different ages are reported in Table 1.

**Figure 2:**
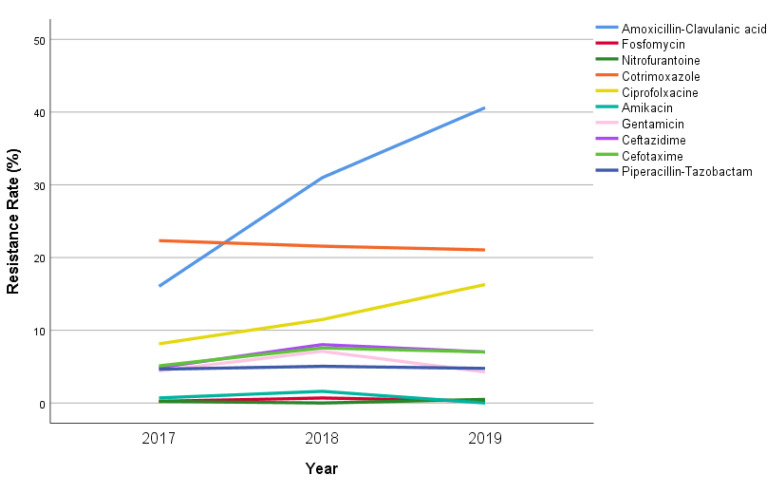
Annual antibiotic resistance rate (% of all isolates) of *E. coli* in our study population.

Considering the incidence by year (Figure 2), the AMC resistance rate significantly increased from 64/363 (17.6%) in 2017 to 131/326 (40.2%) in 2019 (*p* < 0.001). Even the resistance rate for ciprofloxacin doubled in the study period, from 9.1% in 2017 to 16.3% in 2019 (*p* = 0.007). The rates of resistance to cotrimoxazole, cefotaxime, ceftazidime and piperacillin-tazobactam remained almost stable, varying between 2017 and 2019 from 86/363 (23.7%) to 72/326 (22.1%, *p* = 0.852), from 21/363 (5.8%) to 22/326 (6.7%, *p* = 0.625), from 19/363 (5.2%) to 22/326 (6.7%, *p* = 0.628), and from 21/363 (5.8%) to 18/326 (5.5%, *p* = 0.884), respectively.

Moreover, ESBL, MDR, and XDR strains were found in 64 (6.1%), 66 (6.3%), and 14 (1.3%) cases, respectively.

**Figure 3:**
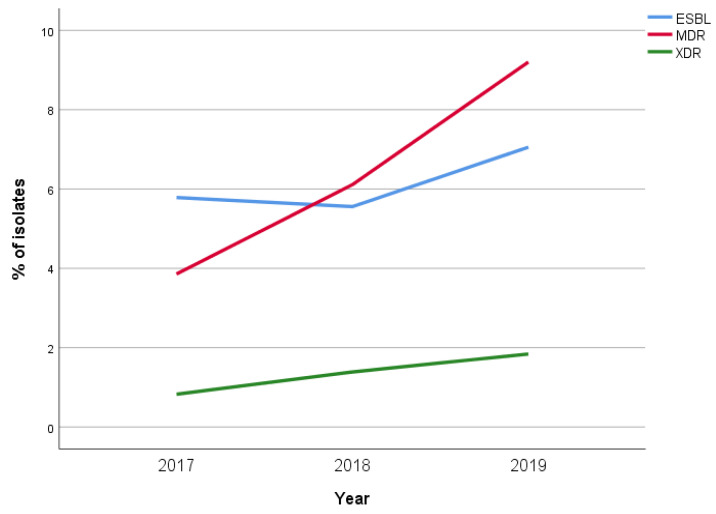
Annual prevalence of ESBL, MDR, and XDR *E. coli* in our study population.

Interestingly, a significant increase in MDR *E. coli* was documented over the study period, rising from 14/363 (3.9%) isolates in 2017 to 30/326 (9.2%) isolates in 2019 (*p* = 0.015). A slight increase in EBSL and XDR isolates was also documented, but the differences were not statistically significant (Figure 3).

## 4. Discussion

Our study showed a 2.3-fold increase (from 17.6% to 40.2%), over a span of three years, in the resistance rate of *E. coli* to AMC in pediatric UTIs in a metropolitan area in northern Italy. To the best of our knowledge, this is the first study to evaluate the trend of resistance rates of *E. coli* to AMC in a large metropolitan area, which includes UCs from both community and hospital patients. The collection of all UCs, regardless of the collection method used and the diagnosis of UTI, was intentionally performed to extensively evaluate the prevalence of AMC-resistant *E. coli* strains circulating in our large metropolitan area.

The high increase in the rate of *E. coli* resistance to AMC could be related to the wide use of AMC as an empirical treatment for several community-acquired infections and for prophylaxis in patients with recurrent UTIs or high-grade vesicoureteral reflux [11]. The annual Emilia-Romagna regional report on antibiotics use and resistance in the pediatric age population documented that 44.1%, 40.0% and 40.5% of children under 14 years of age received at least one antibiotic prescription during the calendar years 2016, 2017, and 2018, respectively [12]. Concurrently, a high, although slowly decreasing, prescription rate of AMC (32.3%, 31.5%, and 30.9% among all antibiotic prescriptions in 2016, 2017, and 2018, respectively) was recorded in the metropolitan area of Bologna [12]. These data could explain the rapid increase in resistant *E. coli* in our population.

The pattern of antibiotic resistance in *E. coli* guides the choice of the empirical treatment of suspected UTIs in children. AMC is indicated as first-line treatment in febrile UTIs by several guidelines, including recently published Italian recommendations [4,11]. This has possibly been suggested by the increasing incidence of unprotected aminopenicillins-resistant strains worldwide and the need to limit the use of second-line antibiotics. Previous studies conducted on Italian cohorts showed a limited rate of *E. coli* resistance to AMC (up to 15%) [8]. Similarly, the Global Report of Surveillance on Antimicrobial Resistance, published in 2014 by the World Health Organization, focused on the resistance of *E. coli* to third-generation cephalosporins and fluoroquinolones, and did not mention AMC-resistant *E. coli* [13]. In 2016, the rate of Gram-negative resistance to oral antibiotics in a single Italian pediatric center was reported in a retrospective analysis conducted by Calzi et al. [14]. This study showed a significant increase in the resistance of *E. coli* to AMC from 23% in 2007 to 36% in 2014.

More recently, several studies have reported an increasing rate of *E. coli* resistance to AMC worldwide. Vihta et al. investigated the incidence of *E. coli* bloodstream infections, UTIs, and antibiotic susceptibilities in Oxfordshire in adults [15]. The authors reported a consistent increase, of 14-29% per year, in the incidence of AMC-resistant *E. coli* among UTIs across the study period (1998–2016). Similarly, Chakupurakal et al. documented a rising resistance pattern of *E. coli* to AMC—from 0% in 2002 to 48% in 2008—in children diagnosed with UTIs in Staffordshire [16].

The mechanism of *E. coli* resistance to AMC seems to be a multifactorial process resulting from combinations of multi-copy beta-lactamase genes, mutations in resistance gene-associated promoters, and inhibitor resistance mechanisms [16]. The hyperproduction of beta-lactamase genes seems to be the most frequent genetic finding, even though there are multiple other mechanisms that regulate the phenotype of *E. coli*. These mechanisms are small and variable, but their effects are additive, resulting in shifts around the clinical breakpoint [17,18]. Therefore, the antibiotic pressure may have induced multiple additional resistance mechanisms, resulting in a rapid increase in the rate of resistance to AMC, in particular involving *E. coli* strains close to the breakpoint. This may have a relevant effect on the therapeutic management of UTIs in children.

At the same time, the emerging problem of high percentages of MDR *E. coli* strains is raising concern among clinicians worldwide [19,20,21]. Our data highlight an increasing incidence of MDR *E. coli*, consistent with the findings of previous studies [18,19,20]. These findings may be associated with the significant rise of AMC resistance. Since resistance is generally mediated by a plasmid encoding resistance to several antimicrobials, this could explain our findings, as previously documented in ESBL-producing bacteria [22].

Host immune responses are critically important in the antimicrobial defense of the urinary tract [23]. *E. coli* interacts with the host immune system cells, in particular with macrophages. To date, it is not clear whether *E. coli* strains with an antibiotic resistance have any advantages or disadvantages in the context of an interaction with the immune system. A longer survival of commensal bacteria carrying specific resistance mutations in the intracellular environment of phagocytes was reported [24].

Several risk factors were described for MDR UTIs, such as underlying urinary tract anomalies, previous hospitalization within the last three months, and previous antibiotic use (including both therapy and prophylaxis). The latter could have played a key role in our area, as suggested by already-mentioned data regarding antibiotic prescriptions in Emilia-Romagna [11]. The role of previous exposure to AMC as a risk factor for *E. coli*-resistant infections was extensively documented by Matanovic et al., who showed a decrease in *E. coli* resistance to the drug, from 37% to 11%, when the overall use of AMC dropped from 30% to 4% in their department [25].

The current prevalence of resistance in our population calls into question the use of AMC as a first-line treatment for UTIs, despite the recent indications of Italian guidelines. Precluding AMC limits the choice of an oral antibiotic as a first-line therapy; therefore, the close monitoring of a child’s condition should be considered until an antimicrobial susceptibility testing result is available or clinical improvement is apparent. Second-generation (e.g., cefuroxime) and third-generation cephalosporin (e.g., cefixime, cefdinir, and ceftibuten) can be considered as possible alternative treatment options for UTIs in children. This choice is guided by the inadequacy of several treatments for UTIs in children (the low safety profile of fluoroquinolones, the difficult handling of aminoglycosides in children, the inadequate tissue concentrations of nitrofurantoin, and the lack of evidence on fosfomycin in pediatric patients) and by the high predicted probability of resistance to some antibiotics (e.g., first-generation cephalosporins, trimethoprim-sulfamethoxazole, and amoxicillin). Nonetheless, the use of third generation cephalosporins may induce additional resistances (e.g., ESBL and MRSA), with further limitations to therapeutic options for UTIs in children.

Our study has some limitations. One key limitation is the lack of data on the past medical history of patients—in particular, on whether the child had previous positive UCs, the presence or absence of congenital anatomic anomalies of the genitourinary tract, and a history of recent hospitalization or antibiotic therapy. Hence, we could not perform any analysis on the possible risk factors for the development of AMC resistance. Moreover, we included urine cultures with a cut-off for colony-forming units lower than the one determined by the American Academy of Pediatrics guidelines [3]. This decision was intended to analyze the prevalence of resistant bacteria in the pediatric UCs without considering a full diagnosis of urinary tract infection.

A further limitation is that we did not investigate children’s clinical response to AMC in order to assess in vivo rather than in vitro resistance, which could be lower than expected.

Furthermore, the resistance rate was defined using the generic AMC clinical breakpoint (S <= 8 mg/L, R > 8 mg/L), as it was not possible to discriminate between complicated and uncomplicated UTIs. Moreover, we did not evaluate the accurate MIC breakpoint/MIC value, which could suggest a different in vivo response to the specific antibiotic.

Larger, multicenter studies are needed to confirm our findings. Directions in future research should include the investigation of risk factors for UTIs caused by AMC-resistant *E. coli* and the assessment of the clinical response of those patients diagnosed with an AMC-resistant *E. coli* UTI and treated with AMC, in order to differentiate in vitro and in vivo resistance.

## 5. Conclusions

Despite the guidelines’ recommendations, our findings give rise to doubts about the appropriateness of AMC prescription and consumption in our metropolitan area and support future investigations aimed at reducing inappropriate area-based antibiotic use. This emerging problem concerning the rising rate of AMC resistant *E. coli* suggests that changing the first-line therapy for UTIs in children is necessary in our area.

## Figures and Tables

**Table 1:** Resistance rate of *E. coli* among different ages.

	<3 Years		3–5 Years		6–10 Years		>10 Years		Total	
	M (*n* = 309)	F (*n* = 378)	M (*n* = 39)	F (*n* = 123)	M (*n* = 33)	F (*n* = 115)	M (*n* = 9)	F (*n* = 43)	M (*n* = 390)	F (*n* = 659)
AMC—*n* (%)	105 (34.0)	97 (25.7)	17 (43.6)	26 (21.1)	13 (39.4)	24 (20.9)	5 (55.6)	9 (20.9)	140 (35.8)	156 (23.6)
FOS—*n* (%)	0 (0.0)	0 (0.0)	0 (0.0)	2 (1.6)	2 (6.1)	0 (0.0)	0 (0.0)	2 (4.7)	2 (0.5)	4 (0.6)
NFT—*n* (%)	0 (0.0)	1 (0.3)	0 (0.0)	0 (0.0)	1 (3.0)	1 (0.9)	0 (0.0)	0 (0.0)	1 (0.3)	2 (0.3)
T/S—*n* (%)	49 (15.9)	90 (23.8)	13 (33.3)	35 (28.5)	13 (39.4)	25 (21.7)	3 (33.3)	10 (23.3)	78 (20.0)	160 (24.3)
CIP—*n* (%)	32 (10.4)	39 (10.3)	7 (17.9)	18 (14.6)	7 (21.2)	13 (11.3)	0 (0.0)	6 (14)	46 (11.8)	76 (11.5)
AMK—*n* (%)	3 (1.0)	2 (0.5)	0 (0.0)	1 (0.8)	0 (0.0)	0 (0.0)	0 (0.0)	0 (0.0)	3 (0.8)	3 (0.5)
GEN—*n* (%)	13 (4.2)	18 (4.8)	3 (7.7)	4 (3.3)	4 (12.1)	3 (2.6)	1 (11.1)	2 (4.7)	21 (5.4)	27 (4.1)
CAZ—*n* (%)	15 (4.9)	22 (5.8)	4 (10.3)	8 (6.5)	4 (12.1)	6 (5.2)	0 (0.0)	1 (2.3)	23 (5.9)	37 (5.6)
CTX–*n* (%)	15 (4.9)	23 (6.1)	4 (10.3)	7 (5.7)	5 (15.2)	6 (5.2)	0 (0.0)	1 (2.3)	24 (6.2)	37 (5.6)
PIT—*n* (%)	31 (10.0)	16 (4.2)	4 (10.3)	1 (0.8)	4 (12.1)	5 (4.3)	0 (0.0)	1 (2.3)	39 (10.0)	23 (3.5)

AMC: amoxicillin/clavulanic acid; FOS: fosfomycin; NFT: nitrofurantoin; T/S: trimethoprim/sulfamethoxazole; CIP: ciprofloxacin; AMK: amikacin; GEN: gentamicin; CAZ: ceftazidime; CTX: cefotaxime; and PIT: piperacillin/tazobactam.

## Data Availability

The data presented in this study are available upon request from the corresponding author.
